# Are current Polish guidelines for prophylactic mastectomy sufficient?

**DOI:** 10.1186/s13053-026-00338-x

**Published:** 2026-06-04

**Authors:** Adam Stachowski, Cezary Cybulski, Jacek Gronwald, Tomasz Huzarski, Tadeusz Dębniak, Tomasz Byrski, Steven Narod, Rodney Scott, Jan Lubiński

**Affiliations:** 1 Department of Genetics and Pathology, International Hereditary Cancer Center, Pomeranian Medical University, ul. Unii Lubelskiej 1, Szczecin, 71-252 Poland; 2 Oncology and Chemotherapy Clinic, University Clinical Hospital no. 2 of the Pomeranian Medical University in Szczecin, al. Powstańców Wielkopolskich 72, Szczecin, 70-111 Poland; 3 Women’s College Research Institute, Women’s College Hospital, University of Toronto, Toronto, ON M5G 1N8 Canada; 4 Centre for Cancer Detection and Therapy, Hunter Medical Research Institute, School of Biomedical Sciences and Pharmacy, Faculty of Health and Medicine, University of Newcastle, Newcastle, NSW 2308 Australia; 5 Division of Molecular Medicine, Pathology North, John Hunter Hospital, New Lambton, NSW 2305 Australia

## Abstract

**Aim:**

Due to the expansion of knowledge about genetic predispositions to breast cancer, the International Hereditary Cancer Center in Szczecin has been solicited to update the Polish ministerial guidelines for the qualification of patients who should be offered prophylactic mastectomy.

**Methods:**

This publication is a review of the scientific literature on the impact of TP53, PTEN, PALB2, CHEK2 pathogenic variants (PVs) predisposing to breast cancer. The number of prophylactic procedures was estimated in May 2024 based on the analysis of pedigree, clinical data and a telephone survey of unselected carriers of the Cancer Genetics Outpatient Clinics in Szczecin.

**Results:**

The literature data justifies prophylactic mastectomy in all women who carry germline pathogenic variants in TP53, PTEN or PALB2. For CHEK2 carriers prophylactic mastectomy is only justified for women carrying a high-risk variant (i.e. carriers of protein-truncating variants with a positive breast cancer family history). We propose these changes will result in approximately 122 additional bilateral procedures and 25 unilateral procedures annually.

**conclusions:**

Current, evidence based data supports extending the qualification criteria for prophylactic mastectomy among TP53, PTEN, PALB and CHEK2 PV carriers.

With 20,000 new cases per year, breast cancer is the most frequent malignancy among women in Poland [1]. The annual costs of treatment are almost 3 billion PLN and increasing [2]. In order to slow this trend, the Polish Ministry of Health refunds surgical prevention for patients, who match the current criteria. This includes women with a strong familial breast cancer history or the documented carrier status of BRCA1/2 pathogenic or likely pathogenic variants (PV) [3]. However, constant progress in medical research has identified many other groups of patients that are at high risk of developing breast cancer and require access to prophylactic mastectomy (also referred to as risk-reducing mastectomy) as well. For this reason, the International Hereditary Cancer Center in Szczecin has solicited a re-appraisal of the current guidelines. The proposal to include additional criteria are as follows:

- Documented carriers of TP53 PVs regardless of cancer family history. These patients are at extremely high risk of developing malignancies that include i.e. sarcomas, adrenal cortical cancers and brain tumors. Breast cancer affects up to 85% of female TP53 PV carriers with a median of age of onset of 34 years [4]. The diagnosis of a pathogenic germline change in TP53 substantially influences treatment choice as well. Oversensitivity to radiotherapy (a significant concern due to secondary tumor initiation in the radiation field), similarly genotoxicity of anthracyclines, cyclophosphamide and platinum-based drugs deprives these patients access to several chemotherapeutic agents [5]. Overall, TP53 PV carriers have worse breast cancer-specific survival than non-carriers (HR = 2,52) [6].
- Documented carriers of PTEN PVs regardless of cancer family history. The burden of such a diagnosis is striking due to an extensive role of the PTEN protein on cell function and DNA integrity. Affected individuals usually develop various dysmorphic features, including macrocephaly, trichilemmomas, hemangiomas, acral keratosis as well as numerous malignancies. One of the most frequent cancers diagnosed in women is breast cancer, which occurs in up to 80% of all PTEN PV carriers [7, 8].
- Documented carriers of PALB2 PVs regardless of cancer family history. PALB2 PVs increase the risk of breast cancer to about 50% [9]. Moreover, PALB2 PVs are associated with the clinical outcome of these patients. According to data reported from our Center the adjusted hazard ratio (HR) for breast cancer-specific death among affected carriers is 2,27, which is dependent on tumor size. The 10-year survival rate drops to just 30% in those with tumors ≥ 20 mm of diameter [10]. These results are consistent with a Finnish report that appeared several years earlier[11] and a study published by Breast Cancer Association Consortium (BCAC) members [6].
- Documented carriers of **high risk** CHEK2 PV. The most prevalent changes in Poland are three founder protein truncating variants (PTV); c. 1100delC, c. 444 + 1G > A, del 5395), where the HR for breast cancer is dependent on family history. In carriers without a breast cancer family history the HR is 3,3 and peaks at about 5, when there is one case among 1st or 2nd degree relatives. In families with greater aggregations of breast cancer the HR can be as high as 7. Interestingly, according to Cybulski *et al*. carriers of PTVs do not have a greater risk of contralateral breast cancer [12]. This finding appears to be a characteristic of the Polish population as Yadav *et al*., who analyzed a cohort of 140 American CHEK2 PTV carriers, found that among ER-positive cancer cases (predominant subgroup) the HR for contralateral breast cancer was 1,9 [13]. A cohort of 656 PTV carriers selected from 34401 women of European ancestry was reported by BCAC[6] where they found a HR for contralateral breast cancer to be 2,25. However, with less than 10% of Poles in the initial group of 34401 the results cannot be extrapolated to the Polish population with the same certainty as those reported by Cybulski *et al*. The question to ask is if there are other CHEK2 PV that need to be included in the guidelines? There is evidence that biallelic CHEK2 PV carriers are at high risk of disease, regardless of cancer family history. The first ever report from our Center showed overall that the HR was 3,9. However if one of the alleles was a PTV, HR peaked at 7. Unfortunately, this report had many limitations due to low study numbers [14]. Currently the largest cohort of 294 biallelic PV carriers (including 212 from Poland) has been described by ERN Genturis [15]. Several years of observation of this population will provide answers to these issues. For the time being, based on reliable scientific evidence, we strongly recommend prophylactic mastectomy only for patients with CHEK2 PTV who have reported that at least one first- or second-degree relative has been diagnosed with breast cancer.

After having selected the population of women who would benefit from surgical intervention, we estimated the number of additional mastectomy procedures required if our proposal is accepted.

For TP53 and PTEN PV carriers the numbers are negligible. Since 1990 the International Hereditary Cancer Center in Szczecin has diagnosed, on average, 1 germline pathogenic change of either gene per year.

For CHEK2 evaluation in May 2024 we examined a sample of 159 CHEK2 PTV carriers diagnosed after 01.01.2019 in Szczecin. We analyzed pedigrees and excluded 27 women that already fitted the current mastectomy qualification criteria. Similarly, 78 women, who had a negative breast cancer family history and one deceased patient. We contacted the remaining patients and asked them if they considered preventive mastectomy. They were all informed about the statistical background of our questionnaire and that their answer would not be considered definitive. Out of 53 patients, who were not excluded in the first phase of the study, we contacted by telephone 41 people. Among these 2 had already undergone unilateral therapeutic mastectomy due to breast cancer (CHEK2 PTV carriers do not have a greater risk of contralateral breast cancer. Therefore, we excluded these patients as there is no benefit from prophylactic contralateral mastectomy), 3 had already undergone preventive mastectomy at their own cost, 9 refused to have mastectomy and 10 did not answer. We identified 17 eligible patients who had never been diagnosed with breast cancer and supported having a bilateral mastectomy. We conclude that 14% of unselected CHEK2 PTV carriers require bilateral prophylactic mastectomy.

For PALB2 the process of evaluation was similar. Apart from not excluding patients with a negative cancer family history our procedure was the same. In May 2024 we analyzed pedigrees of 130 PALB2 carriers diagnosed after 01.01.2015. 24 patients already fitted the criteria, 4 had undergone therapeutic bilateral mastectomy and 4 were deceased. From the remaining 98 women, 91 patients were identified. Of these, 8 had undergone prophylactic mastectomy at their own cost, 27 refused mastectomy and 14 did not answer. The remaining 42 women supported the idea. 12 required unilateral surgery (we support contralateral prophylactic mastectomy in PALB2 PV carriers), while 30 – required bilateral mastectomy. We conclude that 27% of unselected PALB2 PV carriers require prophylactic bilateral mastectomy and 14% need contralateral preventive surgery.

Currently, the International Hereditary Cancer Center in Szczecin systematically collects data on patients from Szczecin and cooperating facilities in i.a. Koszalin, Zielona Góra, Opole, Olsztyn, Rzeszów and Brzozów. It should be emphasized that there are no other major onco-genetic clinics in the vicinity of these cities. Therefore, it can be assumed that the number of new PV diagnoses from these centers reflects data for the whole voivodeships the facilities are located in. Approximately 18.7% of the Polish population lives in the aforementioned regions. We prepared a list of all women with a newly diagnosed CHEK2 PTV and PALB2 PV from these facilities captured between 01.01.2016 and 31.12.2023. We obtained the results of 813 CHEK2 and 242 PALB2 PV carriers. This equates to approximately 550 new carriers of CHEK2 PTV and 162 new carriers of PALB2 PV diagnosed in Poland annually. Based on the evaluation presented above, the new Guidelines would involve approximately:

- for PALB2 PV - an additional 25 unilateral and 45 bilateral mastectomy procedures
- for CHEK2 PTV - an additional 77 bilateral procedures

We estimate that our proposal will result in an additional 122 bilateral and 25 unilateral procedures annually. However,this number may be higher in the first year due to the influx of patients who have been previously diagnosed.

The question is whether prophylactic mastectomy in these groups of women will be cost effective? At the time of writing, a unilateral mastectomy is priced about 20,000 PLN by the National Health Fund and a bilateral procedure about 30,000 PLN. To our knowledge, the efficacy of prophylactic surgery in patients with causative variants in PALB2 or CHEK2 has not been studied. However, prophylactic mastectomy should be as effective as it is for BRCA1 PV carriers, where the residual risk of disease is negligible [16]. Current evidence shows that prophylactic mastectomy also improves overall survival (OS) in initially unaffected BRCA1 PV carriers. The outcomes of prophylactic intervention are highly dependent on the patient’s age at surgery [17]. This is most likely due to women being at high risk of developing ovarian cancer as well. It is ovarian cancer that significantly contributes to premature death and, consequently, the poor OS of the carriers [18]. Ovarian cancer cannot be totally prevented, however, risk-reducing salpingo-oophorectomy, decreases the risk by up to 80% [19]. Nevertheless, in the case of PALB2 PV carriers, the risk of ovarian cancer is only moderately increased and for CHEK2 PTV there is no association [20, 21]. This suggests an even greater benefit on OS for these women. Estimating the impact of preventive mastectomy on OS in PTEN and TP53 PV carriers is very difficult to determine due to the extremely low incidence of carriers in the population, which makes it almost impossible to conduct appropriate prospective studies. In addition, a number of other potentially lethal cancer diagnoses in carriers can lead to premature death despite prophylactic mastectomy.

The group that needs to be analyzed separately are those patients undergoing contralateral prophylactic mastectomy after having been diagnosed with breast cancer (either simultaneously during the therapeutic mastectomy or during a separate operation). In unselected breast cancer patients this procedure reduced breast cancer risk by up to 96%. Nevertheless, OS remained the same [22, 23] or improved negligibly [24]. Among patients with a hereditary breast cancer syndrome, contralateral prophylactic mastectomy decreases the risk of breast cancer similarly [24]. This observation applies to the BRCA1/2 PV carrier population as well [25]. In terms of OS benefits of contralateral prophylactic mastectomy the majority of studies, including a systematic review, show a significant improvement in outcomes [26]. Can these finding be applied to PALB2 PV and CHEK2 PTV carriers? To our knowledge, in these groups the effect of contralateral prophylactic mastectomy on OS has never been reported. However, as the risk of contralateral breast cancer among CHEK2 PTV in the Polish population is not elevated, patients are unlikely to benefit from a prophylactic mastectomy. For PALB2 the risk of contralateral breast cancer is increased. In the Polish cohort 5-year cumulative incidence was as high as 10% [10] and in the study by BCAC, the HR was 2,63 (Morra et al.). According to Yadav *et al.* the risk of contralateral breast cancer depended significantly on estrogen receptor status. For ER-negative patients 10-year cumulative incidence was 19,7%, whereas for ER-positive patients the calculated HR was not statistically significant. Taking these data into account we still recommend contralateral prophylactic mastectomy for PALB2 PV carriers regardless of estrogen receptor status. Although we do not know the impact of surgery on OS in this group, every precaution to reduce the risk of aggressive breast cancer should be applied. For TP53 PV carriers the benefit of contralateral prophylactic mastectomy is undoubted as the risk of contralateral breast cancer is 8 times higher than for non-carriers [6]. For PTEN the 10-year cumulative risk of contralateral breast cancer might be as high as 29% [27]. We therefore suppose that PALB2, TP53, PTEN PV carriers will benefit from prophylactic mastectomy regardless of their oncological history. We may also estimate that in order to avoid one breast cancer we need to perform:

- 2 prophylactic bilateral mastectomies among PALB2 carriers
- 1,7 bilateral mastectomies among CHEK2 PTV carriers with positive cancer family history
- 1,25 bilateral mastectomies among PTEN PV carriers
- 1,18 bilateral mastectomies among TP53 PV carriers

Having defined the cost of mastectomy a comparison with the financial burden of therapy for a single breast cancer can be made. A well-designed estimation was presented by Seweryn et *al*. in 2022, who extracted data from the official databases of the National Health Fund. They analyzed diagnostic and treatment (direct) costs as well as absenteeism (indirect) costs. The overall burden of breast cancer in Poland in 2019 accounted for 344,6 million € (224,3 million € – direct costs and 120,4 million € - indirect cost), which was equal to about 75,000 PLN per patient [28]. However, this value currently appears to be much higher. Recently, many new and expensive pharmacological agents have been introduced. Moreover, in 2021–2023 Poland recorded its highest inflation rate since the early 1990s. In January 2023 the official interest rate peaked at 18,6%, thereby adding to overall costs. Finally, Seweryn *et al.* [2022] emphasized that indirect costs could be five times higher than absenteeism alone. They quoted a robust indirect cost estimation for the years 2010–2014 by Łyszczarz and Nojszewska. In this report other factors, such as: decreased productivity of the patient, disability, premature death, caregivers’ (family members) absenteeism and presenteeism, were taken into account. Patient absenteeism accounted for only 18% of the indirect costs [30]. To our knowledge the latest cost estimation was presented by a large Polish insurance company between the years 2022 and 2023 [2] According to this study, approximately 140,000 PLN was the average cost for a single breast cancer. We do not have access to the report details, however, in the light of considerations described herein this estimate seems plausible. Therefore, we conclude that treatment of a single breast cancer is more than four times more expensive than a single bilateral prophylactic mastectomy.

A comparison of 100 PALB2 PV carriers under surveillance against 100 carriers after prophylactic mastectomy highlights the overall benefits of intervention. Among the 100 PV carriers under surveillance the lifetime unilateral breast cancer risk is 50%. Surveillance will not prevent any cancer, so approximately half of the women will develop disease.

Therefore, what will be the cost of treatment for this group of women? On the one hand it is supposed to be lower than average, due to an earlier diagnosis and on the other hand, PALB2 PV carriers are often diagnosed at a younger age than non-carriers. Consequently, they are more likely to be offered more sophisticated treatment, such as immunotherapy or CDK inhibitors treatments. Because we are unable to include all of these covariates for the purposes of this analysis, we assume that the overall cost in this group will be in line with that reported (i.e ~ 140,000 PLN). We can therefore conclude that in the absence of preventive surgery, it will cost approximately 7 million PLN or even more if we take contralateral breast cancers into account. If the same group undertook prophylactic bilateral mastectomy for 3 million PLN, we would observe only two breast cancer cases at most. It is worth emphasizing that bilateral mastectomy would also greatly reduce annual surveillance costs. With a 1% residual cancer risk it is reasonable to cease high-cost MRI screening. Moreover, lower cancer risk results in fewer control sonographies and biopsies and lower stress levels. For PTEN, TP53 and CHEK2 high-risk PV carriers, cost-effectiveness seems undoubted as the risk of breast cancer is even higher compared to PALB2 PV carriers.

It should be emphasized that our recommendations for PALB2, PTEN, TP53 PV carriers are consistent with the latest NCCN statement presented in the guidelines "Breast cancer risk reduction" [31] and "Genetic/Familial High-Risk Assessment: Breast, Ovarian, Pancreatic, and Prostate "[32]. However, for CHEK2 PTV they have not been adapted to the Polish population. Therefore, the authors base their rather conservative statement on results from international registries, which show 23–27% absolute risk. However, prophylactic mastectomy still may be considered in selected patients, depending on the cancer family history.

In conclusion, there is sufficient evidence that the Ministry's current Guidelines are now due for updating as a result of well-established evidence on the inherited risks of breast cancer. The awareness of the very high risk of breast cancer in carriers of PV in the PALB2, PTEN, TP53 genes should be reflected more adequately in the Guidelines. For CHEK2 PTV, it is justified to introduce the possibility of prophylactic mastectomy in the case of breast cancer in 1st or 2nd degree relatives. Our estimates indicate that there will be approximately 160 additional operations per year, which would have only a modest impact on the workload of the specialist surgical operating theaters. At the same time, preventing malignant tumors in a carefully selected high-risk group of younger women will bring significant savings to the state budget.

## Data Availability

No datasets were generated or analysed during the current study.
